# Efficacy of β-Blockers in Decreasing Mortality in Sepsis and Septic Shock Patients: A Systematic Review

**DOI:** 10.7759/cureus.66888

**Published:** 2024-08-14

**Authors:** Alekya Perala, Annetta V Wishart, Ranim K Hamouda, Entesar Elsaady, Muhammad Rizwan Aslam, Safeera Khan

**Affiliations:** 1 Research, California Institute of Behavioral Neurosciences & Psychology, Fairfield, USA; 2 Internal Medicine, California Institute of Behavioral Neurosciences & Psychology, Fairfield, USA

**Keywords:** septic shock, sepsis, mortality, adrenergic beta-antagonist, β-blockers

## Abstract

Sepsis is a life-threatening condition leading to various organ dysfunction due to an underlying infection. Despite providing appropriate treatment, it is still one of the most common causes of death among patients who are admitted to the intensive care unit (ICU). So, multiple studies have been conducted to identify the potential benefits of various drugs in decreasing mortality in sepsis apart from its traditional treatment options. This study aims to identify whether β-blockers play a role in decreasing mortality in sepsis and septic shock patients because of their potential benefits on several organ systems. Medical databases such as Google Scholar, Summon, PubMed Medical Subject Headings (MeSH), PubMed, Science Direct, Cochrane Library, and Multidisciplinary Digital Publishing Institute (MDPI) were systematically searched for relevant publications. The identified articles were assessed based on the inclusion and exclusion criteria, and 11 research articles were finalized, for which quality appraisal was done using appropriate appraisal tools. β-blockers significantly lowered the in-hospital mortality in sepsis and septic shock patients, and they were also associated with better patient outcomes. As there are limited studies, further research needs to be done to explore the role of β-blockers in decreasing mortality in critically ill populations such as sepsis and septic shock patients.

## Introduction and background

In the landscape of critical care, septic shock remains a challenge that needs to be overcome. Among patients who are admitted to the intensive care unit (ICU), sepsis or septic shock remains the main cause of death. Progression from sepsis to septic shock is characterized by dysfunction of more than two organ systems along with refractory hypotension and increased lactate levels due to tissue hypoperfusion. The underlying pathophysiology consists of marked circulatory, immunologic, hematologic, and metabolic abnormalities, which is the reason for increased mortality [1]. As a physiologic response to the underlying systemic infection and inflammation, there is a widespread activation of the sympathetic nervous system and a surge of catecholamine levels [2]. However, this results in stimulation of β-adrenergic receptors in the heart. The consequence of this β-receptor stimulation is an increase in heart rate, contractility, and cardiac output to cope with the increasing metabolic demands, which can further cause increased stress on the heart and also an imbalance between oxygen supply and demand.

The interplay of these physiologic responses on the heart to the sepsis or septic shock can result in sepsis-induced cardiomyopathy (SIC) [3] and increased incidence of tachyarrhythmias such as sinus tachycardia, atrial fibrillation, and atrial flutter. Tachyarrhythmias are proposed to be an independent risk factor for death or poor prognosis in patients with severe sepsis and septic shock [2-4]. They can further increase the stress on the heart by impairing ventricular filling and increasing myocardial oxygen consumption [3], which in turn can lead to ischemia of the heart. Prolonged catecholamine exposure and excessive activation of the β-receptors are also primary factors linked to heart failure in sepsis [5].

Traditionally, the treatment for septic shock focuses on source control, antibiotic therapy, fluid resuscitation, and vasoactive agents such as norepinephrine (NE) to maintain the mean arterial pressure (MAP) above 65 mmHg. In cases of refractory shock, vasopressin should be combined with NE to reach an acceptable level of pressure control and maintain tissue perfusion. Other supportive measures, such as mechanical ventilation and renal replacement therapy, are also used [4]. Patients with sepsis who continue to have tachycardia even after adequate fluid resuscitation carry a poor prognosis [4]. Therefore, controlling the heart rate might improve the outcomes in the management of sepsis [4].

β-blockers, which are generally used for cardiovascular conditions such as tachycardia, arrhythmias, chronic heart failure, and myocardial infarction (MI) [2], work by reducing heart rate, which can increase the time for diastolic filling and improve the efficiency of the cardiovascular system by maintaining tissue perfusion. Lack of tissue perfusion and tachyarrhythmia, which is a known predictor of poor prognosis in septic shock [6], can exacerbate the ischemia of the heart by causing cardiac dysfunction due to decreased coronary perfusion and increased myocardial oxygen consumption. As β-blocker usage can decrease cardiovascular stress by decreasing heart rate, which can, in turn, protect the already ischemia-prone heart from a secondary MI [7]. So, various studies have been done to identify the beneficial effects of β-blockers in the treatment of sepsis and septic shock patients. Even though β-blockers are proposed to be helpful in septic shock patients by decreasing mortality for all of the above reasons, they can also cause hypotension by decreasing cardiac output [1,2], which can lead to deleterious effects. So, we still need further studies to know which type of β-blocker to use, i.e., short-acting or long-acting, their dosage, how often to give these drugs, and for how long they should be given during the patient's ICU stay.

This study aims to summarize the evidence of the use of β-blockers in decreasing mortality in septic shock patients.

## Review

Methodology

PRISMA 2020 guidelines were used to conduct this systematic review [8].

Search Strategy and Sources Used

We searched PubMed, PubMed Medical Subject Headings (MeSH), Multidisciplinary Digital Publishing Institute (MDPI), Summon, Cochrane Library, Science Direct, and Google Scholar to identify the relevant articles. Table 1 shows the number of papers identified using each database.

**Table 1:** Papers identified using each database. MeSH: Medical Subject Headings

Search strategy	Database used	Number of papers identified
β-blocker AND mortality AND septic shock patients	PubMed	40
(("Adrenergic beta-antagonists/administration and dosage"[Majr] OR "Adrenergic beta-antagonists/pharmacology"[Majr] OR "Adrenergic beta-antagonists/therapeutic use"[Majr])) AND (("Shock, septic/mortality"[Majr] OR "Shock, septic/therapy"[Majr]))	PubMed (MeSH)	37
β-blockers AND mortality AND septic shock	Cochrane Library	10
"β-blockers" AND mortality AND "septic shock"	Science Direct	405
"β-blockers" AND mortality AND "septic shock"	Google Scholar	74
Total		566

Table 2 shows the additional number of papers identified using each database.

**Table 2:** Additional papers identified using each database. MeSH: Medical Subject Headings; MDPI: Multidisciplinary Digital Publishing Institute

Search strategy	Database used	Number of papers identified
(β-blockers[Title/Abstract] AND mortality[Title/Abstract] AND septic shock[Title/Abstract]) AND English [Language]	PubMed	29
("Adrenergic beta-antagonists"[MeSH Terms] AND "Mortality"[MeSH Terms] AND "shock, septic"[MeSH Terms] AND "English"[Language]) AND ((y_10[Filter]) AND (fft[Filter]) AND (humans[Filter]))	PubMed (MeSH)	3
“beta blockers” AND “mortality” AND “septic shock”	MDPI	79
(Abstract:("β-blockers" OR "beta-adrenergic blockers") AND "mortality" AND ("septic shock" OR "sepsis")))	Summon	29
Total		140

Inclusion and Exclusion Criteria

We included articles published in the past 10 years written in English or if the full-text English translation was available. The patient age group included patients above 18 who were admitted to the ICU and satisfied the sepsis or septic shock criteria. For exclusion criteria, articles that were not published in English, patients who were less than 18 years old, and patients who were above 18 years old and admitted to the ICU but did not satisfy the sepsis or septic shock criteria.

Selection Process

The relevant articles were imported to Endnote and duplicate papers were removed. Each qualified article was screened by going through titles and abstracts. After shortlisting the remaining articles, they were assessed for availability of full text. The selected relevant articles with full text were searched for inclusion and exclusion criteria, and articles that did not satisfy these criteria were excluded.

Quality Appraisal of the Shortlisted Articles

The relevant quality appraisal tools were used for all the shortlisted articles based on the type of study conducted. Joanna Briggs Institute (JBI) [9] was used to assess the quality of observational studies, while the Assessment of Multiple Systematic Review (AMSTAR 2) [10] tool was used for systematic review. Narrative reviews were assessed with the Scale for the Assessment of Narrative Review (SANRA) [11], and for randomized controlled trials (RCT), the Cochrane risk-of-bias tool for randomized trials (RoB 2) [12] was used. Only articles that met the quality appraisal criteria were included in this systematic review.

Process of Collecting Data

After the articles were selected for the systematic review, the primary outcome assessed was the mortality benefits of β-blocker usage in sepsis and septic shock patients, along with other additional information such as their protective effects on various organ systems and their role in in-hospital outcomes.

Results

Study Identification and Selection

**Figure 1:**
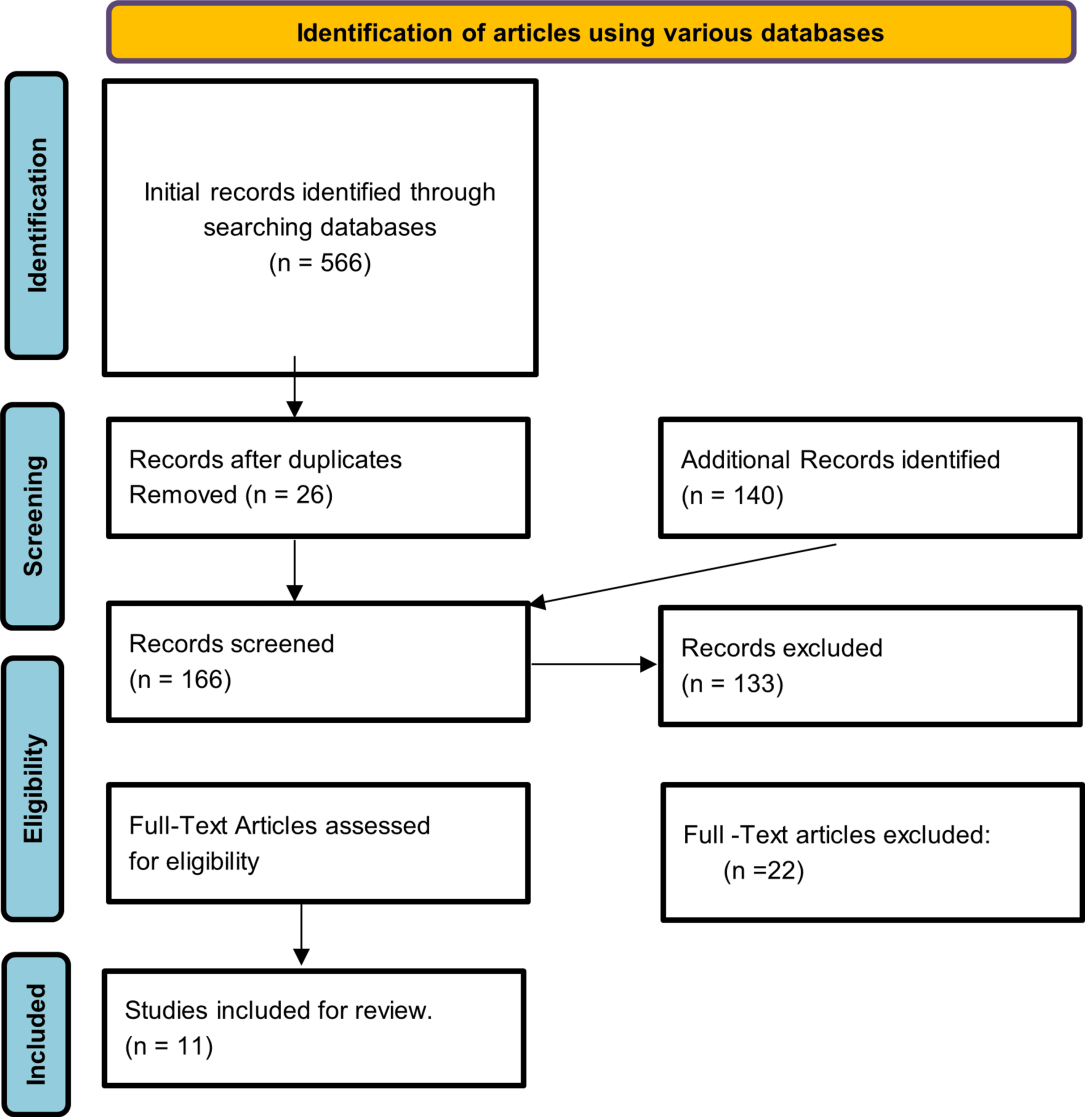
The PRISMA flowchart showing the article selection process. PRISMA: Preferred Reporting Items for the Systemic Review and Meta-Analysis

A total of 566 relevant articles were identified using databases such as PubMed, PubMed (MeSH), MDPI, Summon, Cochrane Library, Science Direct, and Google Scholar. In total, 540 unique duplicate articles were removed before being reviewed in detail. Further search was done to find only articles that were in English. As a result, 140 additional articles were identified. After reviewing these articles in detail by going through the titles and abstracts, 33 articles were shortlisted. The articles that were shortlisted were assessed for eligibility and quality using relevant quality appraisal tools, and a total of 11 articles were finalized for in-depth review. Figure 1 represents the Preferred Reporting Items for the Systemic Review and Meta-Analysis (PRISMA) flowchart, which shows the selection process of all these articles.

The finalized articles were assessed using the relevant quality appraisal tools. Table 3 shows the quality appraisal results using SANRA.

**Table 3:** Quality appraisal using SANRA. SANRA: Scale for the Assessment of Narrative Review

SANRA	Cruz et al. [6]	Fuchs et al. [13]
Importance for the readership justified	2	2
Question formulation and aims stated	2	2
Search for literature described	1	1
Referencing	2	2
Scientific reasoning	2	2
Appropriate presentation of data	1	2

Table 4 shows the quality appraisal results using the AMSTAR tool.

**Table 4:** Quality appraisal using the AMSTAR tool. +: Yes; -: No; AMSTAR: Assessment of Multiple Systematic Review

AMSTAR 2 criteria	Li et al. [5]	Liu et al. [14]
Population, intervention, comparison, and outcome (PICO) components	+	+
Pre-established review methods and any substantial protocol deviation	-	-
Justification for selection of study designs	+	+
Search strategy for the literature explained	+	+
Duplicate study selection performed	+	+
Duplicate data extraction was performed	+	+
Justification for the excluded studies provided	-	+
Detailed description of the included studies	+	+
Assessment of the risk of bias (RoB) in individual studies	+	+
Reporting on the funding sources	-	-
Appropriate methods used for statistical combination of results	+	+
Impact of RoB in individual studies on the results of the meta-analysis	-	-
RoB used in interpreting the results	+	+
Explanation of heterogeneity in the results	+	+
Investigation of publication bias and its impact on the results	-	-
Conflict of interest and funding	+	+

Table 5 shows the quality appraisal results using the JBI tool.

**Table 5:** Quality appraisal using the JBI tool. +: Yes; -: No; ?: Unclear; N/A: Not applicable; JBI: Joanna Briggs Institute

JBI critical appraisal	Yang et al. [2]	Ge et al. [4]
Were the two groups similar and recruited from the same population?	+	+
Measured exposures similarly to assigning people to both exposed and unexposed groups	+	+
Measured exposure validly and reliably	+	+
Identified any confounding factors	+	+
Stated about strategies to deal with confounding factors	+	+
Were the groups free of the outcome while initiating the study?	+	+
Outcomes measured validly and reliably	+	+
Was the follow-up time reported and sufficient enough for outcomes to occur?	+	+
Was the follow-up complete, and if not, explain the reasons for the loss to follow-up?	?	N/A
Were strategies to address incomplete follow-up utilized?	?	N/A
Used appropriate statistical analysis	+	+

Table 6 shows the quality appraisal results using the Cochrane RoB 2 tool.

**Table 6:** Quality appraisal using the Cochrane RoB 2 tool. +: Low risk; ?: Some concerns; -: High risk; RoB: Risk-of-bias tool for randomized trials

Study	Randomization process	Deviations from intended interventions	Missing outcome data	Measurement of the outcome	Selection of the reported result	Overall
Wang et al. 2023 [3]	+	?	+	+	+	+
Gadallah et al. 2020 [7]	+	?	+	+	+	+
Matsuda et al. 2020 [15]	+	?	+	+	+	+
Lira et al. 2014 [16]	+	?	+	?	+	+

Table 7 shows the summary of the finalized articles.

**Table 7:** Summary of the finalized studies. MMIC-IV: Medical Information Market for Intensive Care; eICU: Emergency intensive care unit; SIC: Sepsis-induced cardiomyopathy; Std. MD: Standard mean difference; MAP: Mean arterial pressure; CVP: Central venous pressure; ScvO_2_: Central venous oxygen saturation; TnI: Troponin I; NNT: Number needed to treat

References	Year of study	Study design	Participants [n]	Results	Outcomes/Conclusion	Interpretation
Al-Husinat et al. 2023 [1]	2023	Narrative review	1,969,965	Results indicate that β-blockers, aspirin, and heparin may have positive outcomes in reducing mortality. In comparison, statins don’t play a role in reducing mortality.	This study does not support using β-blockers, aspirin, heparin, or statins in the management of sepsis. Yet, β-blockers have been shown to improve hemodynamics and decrease mortality, while supporting its use in selected patients with sepsis because they are also safe for tissue perfusion.	-
Yang et al. 2023 [2]	2023	Cohort study	61,751	The β-blockers used in this study are atenolol, acebutolol, betaxolol, bisoprolol, esmolol, metoprolol, nadolol, and propranolol. In the MMIC-IV database, the rate of in-hospital deaths for patients using β-blockers was 9.9%, while for those not using β-blockers, it was 19.5%. Similarly, in the eICU database, β-blockers were linked to reduced in-hospital mortality.	The in-hospital mortality rate was significantly lower in those who took β-blockers compared to the patients who did not.	-
Wang et al. 2023 [3]	2023	Randomized controlled trial	100	Patients from the esmolol group achieved a desired heart rate of 80-100/min with a lowering of short-term mortality, improved patient hemodynamics, and improved patient outcomes without any worsening of adverse events compared to the conventional treatment group.	In SIC patients, using esmolol to decrease heart rate reduced short-term mortality while not affecting cardiac contractility.	-
Ge et al. 2023 [4]	2023	Propensity score matching (PSM)	12,360	The β-blockers used in this study are short-acting (esmolol) or long-acting (atenolol, metoprolol, nadolol, and propranolol). β-blockers were linked to lower mortality rates at 28 and 90 days. Long-acting β-blockers resulted in improved 28-day survival and 90-day survival. Whereas short-acting β-blockers did not decrease the 28-day and 90-day mortality rates.	β-blockers were linked to lower 28 and 90-day mortality rates in individuals with sepsis and septic shock. Long-acting β-blocker may protect patients with sepsis by lowering mortality rates at 28 and 90 days. But short-acting β-blocker (esmolol) did not lower sepsis mortality.	-
Li et al. 2019 [5]	2019	Systematic review and meta-analysis	363	β-blocker esmolol resulted in a significantly lower 28-day mortality rate compared to the control group. The β-blocker group also had significantly lower heart rates compared to the standard care group.	β-blocker esmolol is safe and effective for lowering 28-day mortality and controlling ventricular rate in sepsis patients after fluid resuscitation. It has no considerable adverse effect on tissue perfusion.	-
Cruz et al. [6]	2016	Narrative review	-	-	Administration of selective β1-blockers may improve heart function, microcirculation, anti-inflammatory, anti-coagulation, and survival benefits. Esmolol treatment resulted in a considerably decreased 28-day mortality rate.	-
Gadallah et al. 2020 [7]	2020	Randomized controlled trial	60	The β-blocker used in this study is esmolol. β-blockers resulted in a considerable reduction in heart rate, 28-day mortality, and ICU stay.	This study supports the use of intravenous β-blockers in sepsis patients by decreasing heart rate without affecting hemodynamics, as well as decreasing 28-day mortality and ICU stay.	-
Fuchs et al. 2015 [13]	2015	Cohort Study	580	The β-blockers used in this study are atenolol, bisoprolol, carvedilol, metoprolol, nebivolol, propranolol, sotalol, and talinolol. Initiating oral β-blocker before and during sepsis reduced 90-day mortality from 42% to 28% (OR 0.52, 95% CI 0.32-0.87) (P < 0.05). The NNT was 6.9 (95% CI 3.93-28.40).	The current findings show that oral β-blockers should not be discontinued in patients with severe sepsis and septic shock. Furthermore, after initial stabilization, these patients should be considered for initiation of a β-blocker therapy.	-
Liu et al. 2018 [14]	2018	Meta-analysis	41 to 154	Esmolol intervention increased survival rate (P=0.006), decreased heart rate (P=0.005), and TnI (P＜0.00001), without any significant effect on mean arterial pressure (MAP) (Std. MD=0.11; 95% CI=-0.21 to 0.44).	Treatment with esmolol can improve survival rates with a reduction of heart rate and TnI levels, without any impact on MAP, CVP, or ScvO_2_ in patients with sepsis and septic shock.	-
Matsuda et al. 2020 [15]	2020	Randomized controlled trial	151	Landiolol, a β-blocker, effectively reduced heart rate to 60-94 beats/min at 24 h, new-onset arrhythmia, and 28-day mortality in most patient categories without significantly increasing the frequency of adverse events.	-	This study demonstrated that the key patient characteristics of most patients have no effect on the efficacy and safety of landiolol.
Lira et al. 2014 [16]	2014	Randomized controlled trial	77 - Esmolol group, 77 - Control group, Total - 154	A targeted heart rate of 82-94 bpm was achieved in all patients in the esmolol group compared to those in the control group. The 28-day mortality rate was 49.4% in the esmolol group and 80.5% in the control group (P < 0.001).	For patients in septic shock, open-label esmolol use versus conventional therapy was associated with lowering heart rates to achieve target levels while avoiding incidents of adverse effects.	-

Discussion

Sepsis and Its Mortality Rate

Despite the advances in recognizing and treating the underlying infections in sepsis, it still has a high mortality rate of 20-30% [2]. Among hospitalized patients, it is the leading cause of death with a 30-40% mortality rate [4]. About 19.7% of global deaths are due to sepsis, which is approximately 49 million cases in 2017 [1]. According to one study, severe septic shock carries a mortality rate of 50-60% in the critically ill population [16]. Sepsis can progress to SIC in 50% of sepsis patients and up to 70% of septic shock patients, which is also one of the causes of the increased rate of mortality [3,14].

Pathophysiology of Sepsis and Its Effect on Various Organ Systems

Sepsis, which stems from an underlying infection, results in excessive vasodilation and exaggerated catecholamine surge. This endogenous catecholamine surge leads to excessive activation of the adrenergic system. This can cause an increase in heart rate, stroke volume, and mean arterial pressure. However, this excessive and continuous activation of the adrenergic system can cause tachyarrhythmias such as atrial fibrillation and atrial flutter that can impair diastolic filling and myocardial perfusion, which can further impair myocardial contractility, resulting in decreased left ventricular ejection fraction. This decrease in ejection fraction leads to oxygen supply and demand mismatch, worsening metabolic acidosis by increasing lactic acid levels. Developing tachyarrhythmias in the setting of sepsis can lead to SIC, which is defined as decreased left ventricular ejection fraction, left ventricular dilation, and complete recovery in 7-10 days [6]. According to a study, the echocardiography shows around 50% of individuals with septic shock develop cardiomyopathy [6]. As all these deleterious effects are caused by continuous stimulation of the adrenergic system, various studies have been conducted to study the effectiveness of β-blockers in decreasing catecholamine release and decreasing sympathetic activity, which can, in turn, improve heart rate and diastolic function, which can be associated with better patient outcomes and mortality rate.

**Figure 2:**
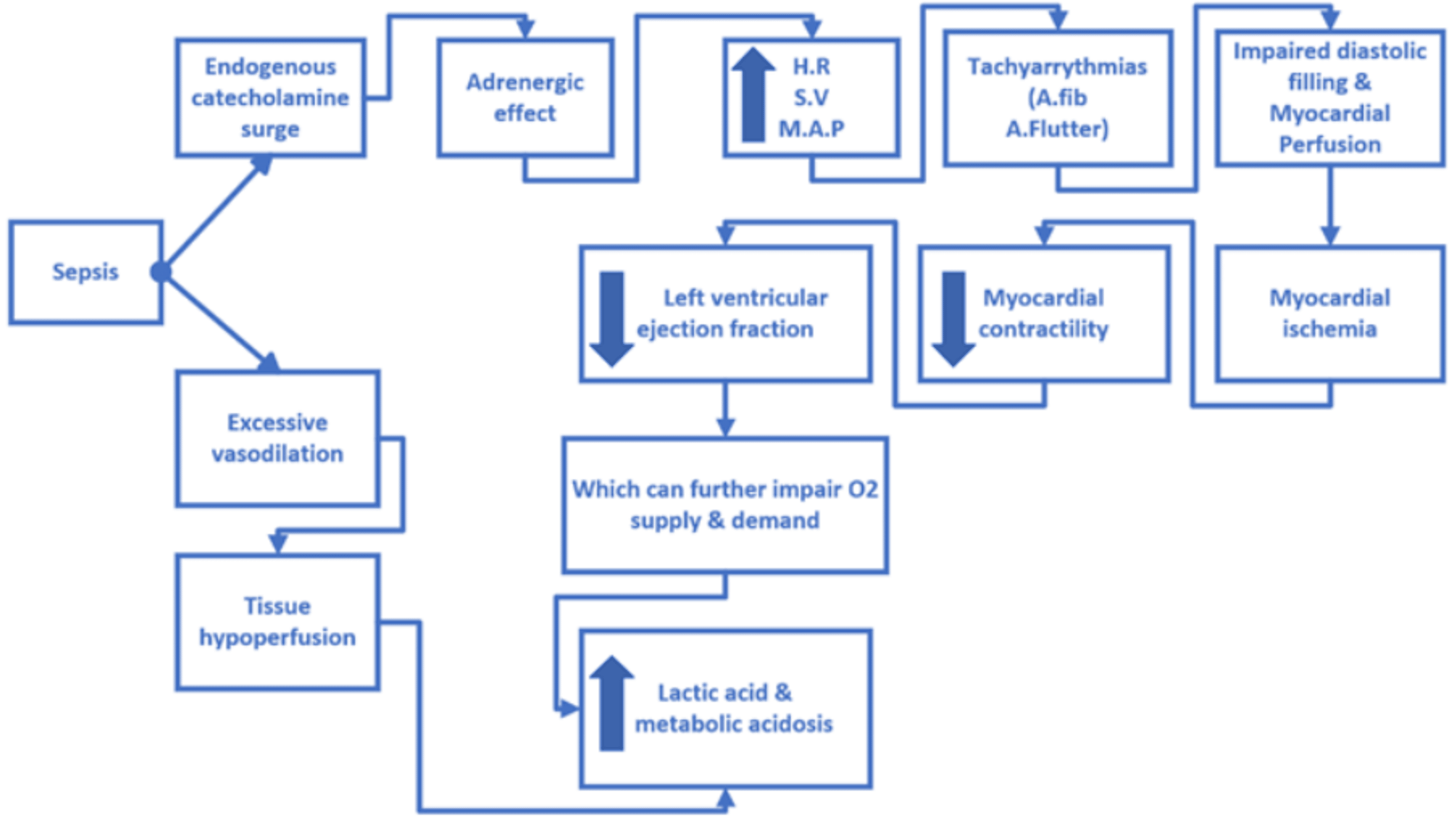
Flowchart of the pathophysiology. H.R: Heart rate; S.V: Stroke volume; M.A.P: Mean arterial pressure; A.fib: Atrial fibrillation; A.flutter: Atrial flutter The figure was created by the author Alekya Perala using Microsoft Visio (Microsoft Corp., Redmond, USA).

Figure 2 shows the pathophysiology of sepsis.

Mechanism of Action of β-Blockers

β-blockers are of two types, namely selective and non-selective. Selective β-blockers such as atenolol, acebutolol, bisoprolol, esmolol, and metoprolol are the ones that block β1-receptors that are found mainly in cardiac nodal tissue, cardiac myocytes, and also on kidneys [17]. Whereas, non-selective β-blockers such as propranolol, nadolol, and sotalol block both β1 and β2-receptors. β2 receptors are found mainly in bronchial and smooth muscles [18].

Circulating catecholamines such as epinephrine and norepinephrine act on β1 and β2-receptors. Both of these are G-protein coupled receptors that work by conversion of adenosine triphosphate (ATP) to cyclic adenosine monophosphate (CAMP) via adenyl cyclase, which in turn leads to an increase in intracellular calcium ion (Ca^2+^). This causes phosphorylation of myosin light chains to cause muscle contraction. Activation of β1-receptors in the heart leads to sinoatrial (SA) node, atrioventricular (AV) node, and ventricular muscle firing that leads to an increase in contractility (inotropy), heart rate (chronotropy), and cardiac conduction timing (dromotropy). Whereas, activation of β1-receptors in the kidneys causes secretion of renin and eventually leads to an increase in the blood volume [17]. β-blockers work by competing with catecholamines such as epinephrine and norepinephrine for β-receptor sites. This blockade leads to a decrease in inotropy, chronotropy, and dromotropy and an increase in lusitropy (relaxation) of the heart [17].

Protective Effect of β-Blockers When Used in Septic Shock

Studies indicate that β-blockers have the potential to lower heart rate and reduce sympathetic activity [2,7]. Berk et al. [19] was the one who first studied the use of β-blockers in 1969 to diminish the sympathetic nerve activation in sepsis, which can be associated with decreased mortality [2]. They can also improve hemodynamics by enhancing stroke index, systemic vascular resistance, and left ventricular stroke work indices (Morelli et al. [20]). Additionally, they play a role in regulating the inflammatory response [2,3] by decreasing inflammatory mediators like interleukin-6 (IL-6), high mobility group box 1 protein (HMGB1), and tumor necrosis factor-alpha (TNF-α) [2] while also preventing cardiomyocyte death. They can also reduce markers of cardiac injury [1] and help preserve myocardial function [14].

Blocking the adrenergic receptor could potentially avoid catecholamine toxicity in cases of septic shock [16]. When β-blockers are paired with standard septic shock treatments, there may be an enhancement in cardiac function and vascular responsiveness to catecholamines, as suggested by Kimmoun et al. [3,21]. Study shows that selective β-blocker esmolol increases oxygen supply while decreasing oxygen consumption and also reduces cardiac myocyte injury markers like troponin I (TnI) levels [14]. All of which can eventually improve patient prognosis and lower mortality rates [3].

Analysis of Various Studies in Decreasing Mortality in Septic Shock by Using β-Blockers

A retrospective cohort study was conducted in patients with sepsis using Medical Information Mart for Intensive Care-IV (MIMIC-IV) and the emergency ICU databases to assess for in-hospital mortality. This study showed that in-hospital mortality was significantly lower in the β-blocker group compared to the non-β-blocker group. The in-hospital mortality for the β-blockers was 9.9%, and the non-β-blocker group was 19.5% [2].

A propensity score matching (PSM) analysis showed that β-blockers were associated with improved 28 and 90-day mortality, especially when long-acting β-blockers were used, whereas short-acting β-blockers did not [4].

A randomized controlled trial that included 100 SIC patients with a heart rate above 100/min showed that the use of esmolol in these patients achieved a target heart rate of 80-100 bpm without decreasing myocardial contractility and worsening adverse events, along with lowering their 28 and 90-day mortality [3].

Similarly, another study showed that inhibition of catecholamine secretion by β-blockers brings down the heart rate to < 95bpm within 24 hours, which can have a positive impact on the patient outcome. Both selective and non-selective β-blockers were reported to reduce mortality along with reducing markers of cardiac injury [1].

Similarly, various studies conducted showed a significant decrease in heart rate [7,15,16] by decreasing sympathetic overactivation, improved tissue perfusion and hemodynamics, along with a decrease in 28-day mortality [2-4,7,16] and a decrease in 90-day mortality [4,6,13]. Additional benefits of β-blockers include reduced length of stay (LOS) in the ICU [1,4,5,7], decrease in vasopressor requirements [1,2], and increase in ventilator-free days [1-3,7]. It has been shown that β-blocker landiolol may improve sepsis-induced acute lung injury through the pulmonary endothelin system [2]. There is also a decrease in serum lactate, central venous-to-arterial carbon dioxide difference (PcvaCO_2_ gap) [1], and improved central venous oxygen saturation (ScvO_2_) [3] with the use of esmolol. Septic patients with continuously low ScvO_2_ imply cardiac dysfunction, which can lead to an increase in 90-day mortality [3]. Because of all these numerous therapeutic benefits of β-blockers, they have the potential to be added to future management of sepsis.

Limitations

Although there are very promising benefits of β-blocker usage in the management of sepsis and septic shock patients including a significant decrease in in-hospital mortality leading to better patient outcomes, the results also highlighted adverse events such as bradycardia, AV-nodal block, and hypotension [4,17]. This raises a concern whether β-blockers are safe to use in hemodynamically unstable patients with sepsis-induced tachycardia. Therefore, further studies need to be done to assess the role of β-blockers in the management of sepsis and septic shock.

## Conclusions

This study suggests that the use of β-blockers in sepsis and septic shock patients is associated with a significant decrease in in-hospital mortality and also associated with better patient outcomes. As β-blockers cause hypotension and can deteriorate the patient’s condition, carefully curated controlled studies are needed to study the dosages, such as loading dose and maintenance dose, and how long they should be given for needs to be studied. Further large-scale studies are needed to add β-blockers to the standard treatment regimen of sepsis and septic shock.

## References

[1] Al-Husinat L, Abu Hmaid A, Abbas H (2023). Role of aspirin, beta-blocker, statins, and heparin therapy in septic patients under mechanical ventilation: a narrative review. Front Med (Lausanne).

[2] Yang Q, Kong T, Bao Z (2023). Association between the β-blocker use and patients with sepsis: a cohort study. Front Med (Lausanne).

[3] Wang J, Gao X, He Z, Wang J, Xu G, Li T (2023). Evaluating the effects of esmolol on cardiac function in patients with septic cardiomyopathy by speck-tracking echocardiography - a randomized controlled trial. BMC Anesthesiol.

[4] Ge CL, Zhang LN, Ai YH, Chen W, Ye ZW, Zou Y, Peng QY (2023). Effect of β-blockers on mortality in patients with sepsis: a propensity-score matched analysis. Front Cell Infect Microbiol.

[5] Li J, Sun W, Guo Y, Ren Y, Li Y, Yang Z (2020). Prognosis of β-adrenergic blockade therapy on septic shock and sepsis: a systematic review and meta-analysis of randomized controlled studies. Cytokine.

[6] Cruz MC, Reis L (2017). β-blockers in septic shock: are we there yet?. Rev Bras Ter Intensiva.

[7] Gadallah RR, Aboseif EMK, Ibrahim DA, Zaki HV, Abdelmaksoud MNM (2020). Evaluation of the safety and efficacy of beta blockers in septic patients: a randomized control trial. Ain-Shams J Anesthesiol.

[8] Page MJ, McKenzie JE, Bossuyt PM (2021). The PRISMA 2020 statement: an updated guideline for reporting systematic reviews. BMJ.

[9] Barker TH, Stone JC, Sears K (2023). Revising the JBI quantitative critical appraisal tools to improve their applicability: an overview of methods and the development process. JBI Evid Synth.

[10] Shea BJ, Reeves BC, Wells G (2017). AMSTAR 2: a critical appraisal tool for systematic reviews that include randomised or non-randomised studies of healthcare interventions, or both. BMJ.

[11] Baethge C, Goldbeck-Wood S, Mertens S (2019). SANRA - a scale for the quality assessment of narrative review articles. Res Integr Peer Rev.

[12] Sterne JA, Savović J, Page MJ (2019). RoB 2: a revised tool for assessing risk of bias in randomised trials. BMJ.

[13] Fuchs C, Scheer C, Wauschkuhn S (2015). 90-day mortality of severe sepsis and septic shock is reduced by initiation of oral beta-blocker therapy and increased by discontinuation of a pre-existing beta-blocker treatment. Intensive Care Med Exp.

[14] Liu P, Wu Q, Tang Y, Zhou Z, Feng M (2018). The influence of esmolol on septic shock and sepsis: a meta-analysis of randomized controlled studies. Am J Emerg Med.

[15] Matsuda N, Nishida O, Taniguchi T (2020). Impact of patient characteristics on the efficacy and safety of landiolol in patients with sepsis-related tachyarrhythmia: subanalysis of the J-Land 3S randomised controlled study. EClinicalMedicine.

[16] Lira A, Pinsky MR (2014). Should β-blockers be used in septic shock?. Crit Care.

[17] Tucker WD, Sankar P, Kariyanna PT (2024). Selective beta-1 blockers. StatPearls [Internet].

[18] (2012). Beta adrenergic blocking agents. LiverTox: Clinical and Research Information on Drug-Induced Liver Injury [Internet].

[19] Berk JL, Hagen JF, Beyer WH, Gerber MJ, Dochat GR (1969). The treatment of endotoxin shock by beta adrenergic blockade. Ann Surg.

[20] Morelli A, Ertmer C, Westphal M (2013). Effect of heart rate control with esmolol on hemodynamic and clinical outcomes in patients with septic shock: a randomized clinical trial. JAMA.

[21] Kimmoun A, Louis H, Al Kattani N (2015). β1-adrenergic inhibition improves cardiac and vascular function in experimental septic shock. Crit Care Med.

